# Appropriate intraprocedural initial heparin dosing in patients undergoing catheter ablation for atrial fibrillation receiving uninterrupted non-vitamin-K antagonist oral anticoagulant treatment

**DOI:** 10.1186/s12872-021-02032-3

**Published:** 2021-04-27

**Authors:** Rong-feng Zhang, Cheng-ming Ma, Na Wang, Ming-hui Yang, Wen-wen Li, Xiao-meng Yin, Ying-xue Dong, Xiao-hong Yu, Xian-jie Xiao, Yun-long Xia, Lian-jun Gao

**Affiliations:** 1 Department of Cardiology, Institute of Cardiovascular Diseases, First Affiliated Hospital of Dalian Medical University, 193# Lianhe Road, Shahekou District, Dalian, China; 2 Department of Ultrasonics, Second Affiliated Hospital of Xi’an Jiaotong University, Xi’an, China; 3 Department of Intensive care medicine, First Affiliated Hospital of Dalian Medical University, 193# Lianhe Road, Shahekou District, Dalian, China

**Keywords:** Atrial fibrillation, New oral anticoagulants, Radiofrequency catheter ablation, Bleeding

## Abstract

**Background:**

To clarify the appropriate initial dosage of heparin during radiofrequency catheter ablation (RFCA) in patients with atrial fibrillation (AF) receiving uninterrupted nonvitamin K antagonist oral anticoagulant (NOAC) treatment.

**Methods:**

A total of 187 consecutive AF patients who underwent their first RFCA in our center were included. In the warfarin group (WG), an initial heparin dose of 100 U/kg was administered (control group: n = 38). The patients who were on NOACs were randomly divided into 3 NOAC groups (NG: n = 149), NG110, NG120, and NG130, and were administered initial heparin doses of 110 U/kg, 120 U/kg, and 130 U/kg, respectively. During RFCA, the activated clotting time (ACT) was measured every 15 min, and the target ACT was maintained at 250–350 s by intermittent heparin infusion. The baseline ACT and ACTs at each 15-min interval, the average percentage of measurements at the target ACT, and the incidence of periprocedural bleeding and thromboembolic complications were recorded and analyzed.

**Results:**

There was no significant difference in sex, age, weight, or baseline ACT among the four groups. The 15 min-ACT, 30 min-ACT, and 45 min-ACT were significantly longer in the WG than in NG110 and NG120. However, no significant difference in 60 min-ACT or 75 min-ACT was detected. The average percentages of measurements at the target ACT in NG120 (82.2 ± 23.6%) and NG130 (84.8 ± 23.7%) were remarkably higher than those in the WG (63.4 ± 36.2%, *p* = 0.007, 0.003, respectively). These differences were independent of the type of NOAC. The proportion of ACTs in 300–350 s in NG130 was higher than in WG (32.4 ± 31.8 vs. 34.7 ± 30.6, *p* = 0.735). Severe periprocedural thromboembolic and bleeding complications were not observed.

**Conclusions:**

For patients with AF receiving uninterrupted NOAC treatment who underwent RFCA, an initial heparin dosage of 120 U/kg or 130 U/kg can provide an adequate intraprocedural anticoagulant effect, and 130 U/kg allowed ACT to reach the target earlier.

*Trial registration*: Registration number: ChiCTR1800016491, First Registration Date: 04/06/2018 (Chinese Clinical Trial Registry http://www.chictr.org.cn/index.aspx).

**Supplementary Information:**

The online version contains supplementary material available at 10.1186/s12872-021-02032-3.

## Background

Atrial fibrillation (AF) is one of the most common clinical arrhythmias, with a prevalence rate of 0.4–2%, and increased with advancing age [1]. AF is associated with a fivefold increased risk of stroke; moreover, AF-related stroke is more severe than non-AF-related stroke [2]. Thus, adequate anticoagulant therapy is essential for patients with AF. Over the past few years, non-vitamin-K antagonist oral anticoagulants (NOACs) have been increasingly widely used and have reduced stroke and systemic embolism compared with warfarin, with a lower bleeding risk [3].

Radiofrequency catheter ablation (RFCA) is a safe and effective alternative method to sustain sinus rhythm and improve symptoms for patients with AF. However, the risk of periprocedural thromboembolism remains an intractable issue, with an incidence rate of 1–5%. The routine application of heparin to maintain an activated clotting time (ACT) of 250–350 s during RFCA was recommended in the Chinese AF treatment guidelines [4]. The Heart Rhythm Society's Scientific Statement suggested 50–100 U/kg of heparin administration before transseptal puncture in patients on warfarin anticoagulants. Compared with patients on warfarin, the average time required to reach the target ACT was longer in patients treated with NOACs, who needed more intraprocedural heparin doses [5]. Nevertheless, few studies focusing on the initial heparin dosage in patients with NOACs have been reported. We compared the actual percentages of measurements at the target ACT with different initial heparin doses in patients taking NOACs and patients taking warfarin to determine the appropriate dosage of initial intraprocedural heparin.

## Methods

### Study design

This study was a single-center, randomized, double-blind prospective clinical trial conducted in our center from 22/06/2018 to 21/05/2019. This trial was registered in Chinese Clinical Trial Registry (Registration number: ChiCTR1800016491, First Registration Date: 04/06/2018), and was approved by the Ethics Committee of the First Affiliated Hospital of Dalian Medical University.

We hypothesized that a 100 U/kg initial bolus of heparin in patients on uninterrupted warfarin treatment would be a reliable control dose based on the Heart Rhythm Society's Scientific Statement. Based on our experience and earlier studies, a 100 U/kg initial bolus of heparin cannot provide adequate intraprocedural anticoagulation in patients with NOACs [5, 6]. Therefore, in the NOAC groups (NGs), we set the initial bolus of heparin as 110 U/kg (NG110), 120 U/kg (NG120), and 130 U/kg (NG130). We compared the actual percentages of measurements at the target ACT in the three NGs with those in the warfarin group to determine a proper dosage of initial heparin for NOACs.

### Study subjects

The detailed inclusion/exclusion criteria are shown in the Additional file 1. A total of 187 consecutive patients aged 18–75 years with nonvalvular AF who underwent RFCA were enrolled in our study. Patients with a past medical history of cardiac surgery or cardiac device implantation, severe hepatic/renal insufficiency, or coagulation disorders were excluded. Patients with a left ventricular ejection fraction (LVEF) ≤ 40% or left atrial diameter (LAD) ≥ 55 mm were not included.

**Fig. 1:**
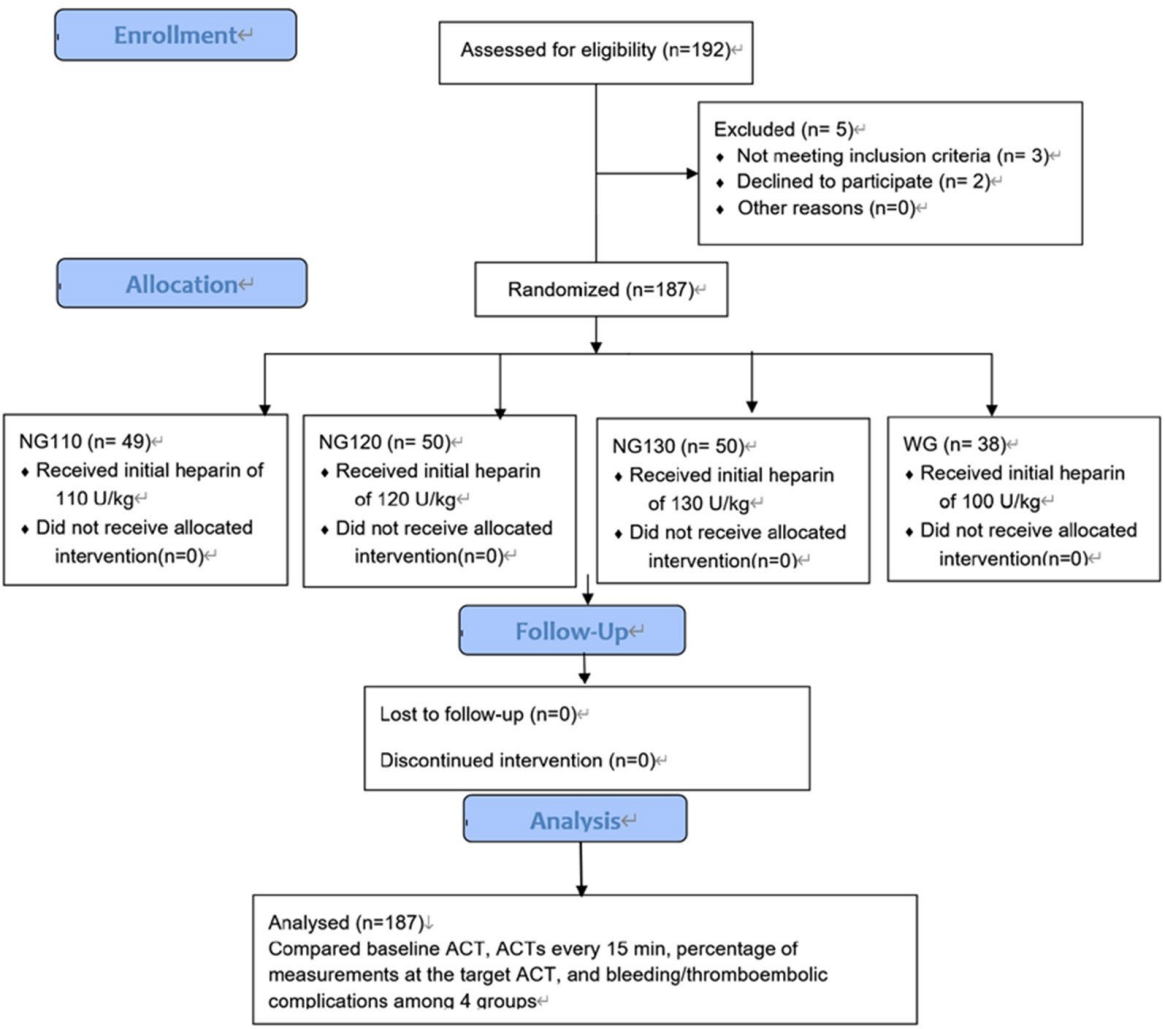
Flow chart of the enrollment and processing of study patients

According to the preprocedural anticoagulant, the 187 patients were classified into a warfarin (control) group (WG: n = 38) and 3 NOAC groups (NG: n = 149). The patients to receive NOACs were randomly divided into three groups by random number generator in SPSS (IBM, Armonk, NY, USA) and were administered three different initial doses of heparin (110,120,130 U/kg) (Fig. 1).

### Preprocedural anticoagulant

For patients with persistent AF or paroxysmal AF with a CHA_2_DS_2_-VASc score ≥ 2, warfarin (international normalized ratio (INR) 2.0–3.0 and the time in range (TTR) > 60%) or NOAC (dabigatran 110 mg twice a day or rivaroxaban 20 mg or 15 mg once a day for patients with a HAS-BLED score ≥ 3 or estimated glomerular filtration rate ≤ 50 ml/min) anticoagulant therapy was administered for at least three weeks prior to ablation. The patients with paroxysmal AF with a CHA_2_DS_2_-VASc score < 2 were anticoagulated with warfarin or NOACs preoperatively. Atrial thrombosis was excluded by pulmonary vein (PV) computed tomography or transesophageal echocardiography within 48 h before the ablation procedure.

### Catheter ablation

During the catheter ablation (CA) procedure, a decapolar catheter was positioned in the coronary sinus for atrial pacing and signal reference via the right femoral vein. Two 8-F long sheaths (SL1, Synaptic Medical, Beijing, China) were delivered into the left atrium using a modified Brockenbrough technique. The transseptal sheaths were flushed continuously with heparinized saline (22 ml/h) to prevent thrombus formation. A circular PV mapping catheter (LASSO, Biosense Webster, Irvine, USA) and a saline-irrigated ablation catheter (Thermocool SMART TOUCH SF, Biosense Webster, Irvine, USA) were applied for mapping and ablation using the CARTO 3 electroanatomic mapping system (Biosense Webster, Irvine, USA).

Continuous ablation was performed alongside each PV antrum to encircle the ipsilateral PVs (target temperature 45 °C, maximum power 35 W, and infusion rate 22 ml/min). The endpoint of RFCA for paroxysmal AF was complete electrical PV isolation as demonstrated by the absence of PV potentials or PV-left atrium conduction. For nonparoxysmal AF, another linear ablation (left atrial roof, mitral isthmus, or tricuspid isthmus) was performed to achieve a bidirectional block or restore sinus rhythm. If the sinus rhythm failed to be converted, we performed direct current electric conversion, followed by the verification of PV isolation and bidirectional block and complementary ablation. If atrial flutter (AFL) had been documented before ablation or was induced during the procedure, the relevant substrate was mapped and ablated.

### Intraprocedural heparin administration

**Fig. 2:**
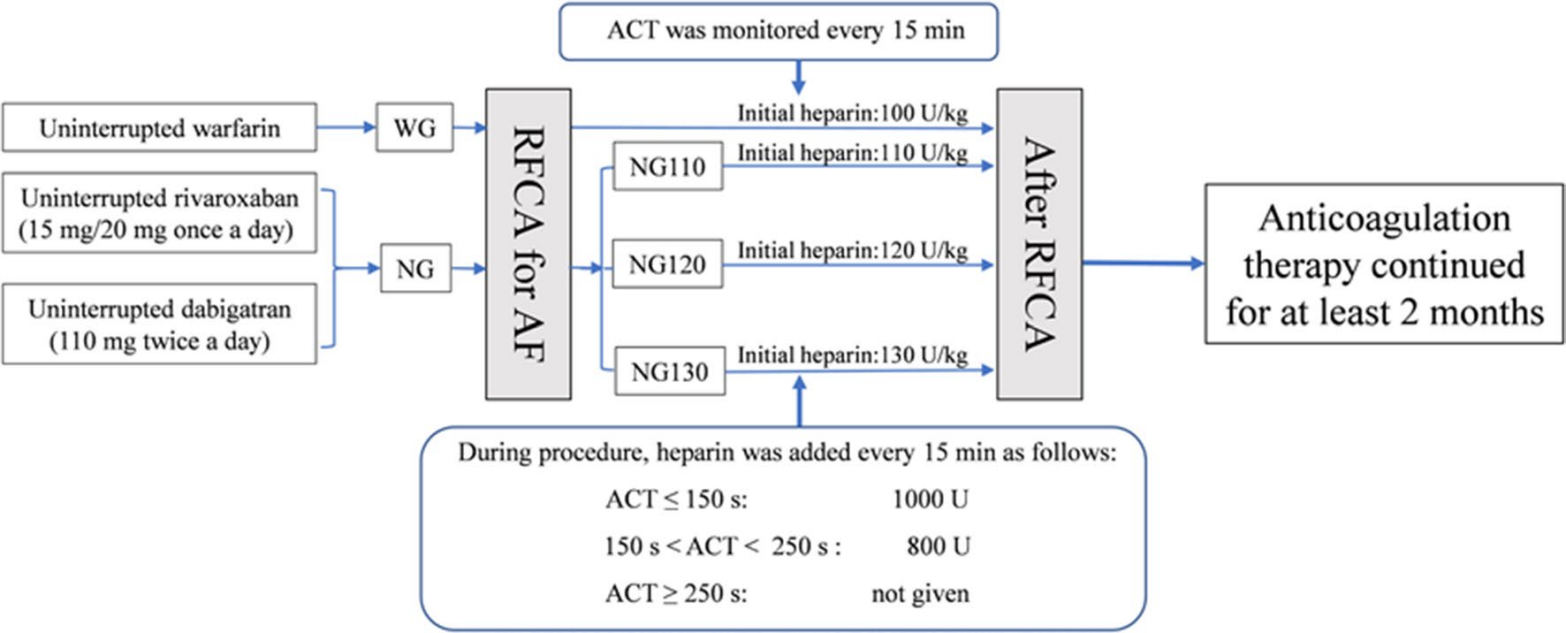
Periprocedural anticoagulation regimens and measurement of ACT during the procedure

Intraprocedural ACT monitoring was carried out by the Automated Coagulation Timer System (ACT Plus, Medtronic, Minneapolis, USA). Patients in the WG were given an initial heparin dose of 100 U/kg. In contrast, patients in the NG were given initial heparin doses of 110 U/kg (NG110), 120 U/kg (NG120), and 130 U/kg (NG130). The baseline ACT was measured before transseptal puncture, and then the intraprocedural ACT was measured every 15 min. The initial dosage of heparin was administered immediately after the transseptal puncture. The supplemental dosage of heparin was determined based on the operator's experience and the measured ACT to maintain ACT between 250 and 350 s. If the measured ACT did not reach the target, an additional dose of heparin was injected immediately according to the regimen as follows: if ACT was 150–250 s, a heparin dose of 800 U was added, if ACT < 150 s, a heparin dose of 1000 U was added. An additional dose of heparin was not given if the ACT reached the target or was ≥ 350 s (Fig. 2). Heparin was discontinued immediately in case of severe bleeding complications, and then protamine sulfate was administrated, followed by urgent pericardiocentesis, blood transfusion, or cardiac surgery if necessary.

The baseline ACT, ACTs at each 15-min interval (15 min-ACT, 30 min-ACT, 45 min-ACT, 60 min-ACT, 75 min-ACT), and the average percentage of measurements at the target ACT were recorded and analyzed.

### Follow-up

The incidence of bleeding and thromboembolic complications was recorded and analyzed. Major bleeding complications included pericardial tamponade, retroperitoneal hemorrhage, hemoglobin drop ≥ 4 g/dL, and the need for blood transfusion. Minor bleeding complications included pericardial effusion, inguinal hematoma, hematuria, or gastrointestinal bleeding. Thromboembolic complications included stroke, transient ischemic attacks (TIAs), deep vein embolism, or pulmonary embolism. The periprocedural complications were defined as adverse events that occurred within 30 days after the procedure.

After the procedure, anticoagulation therapy continued for at least two months. All patients were followed up monthly in the clinic for at least one month post ablation.

### Statistical analysis

All analyses were performed using SPSS version 22.0 (SPSS Inc., Chicago, USA). Continuous variables are expressed as the mean ± standard deviation (SD) if normally distributed; the median and the 25–75% interquartile range were used for skewed data. An unpaired t-test or one‐way analysis of variance was performed for measurement data. For categorical variables, chi-square tests or Fisher's exact tests were used. The ACT levels and the actual percentages of measurements at the target ACT in the three NGs were compared with those in the WG, respectively. A 2-tailed *p* value < 0.05 was considered statistically significant.

## Results

### Baseline clinical characteristics

The baseline clinical characteristics of the WG and NGs are summarized in Table 1. There was no significant difference between the WG and the NGs in age, sex, comorbidity, CHA_2_DS_2_-VASc score, HAS-BLED score, procedure time, ablation time, etc. There was a significant difference in the proportion of patients with nonparoxysmal AF, left ventricular diameter, and weight between the four groups. No significant difference was found in baseline characteristics among the dabigatran groups (DG110, DG120, DG130) or among the rivaroxaban groups (RG110, RG120, RG130) (Additional file 1: Additional file 2, Table 1). In the WG, the average preprocedural INR was 2.2 ± 0.9, and the TTR was 63.16%. In the NGs, patients received dabigatran for 110 mg twice per day or rivaroxaban for 15 mg or 20 mg once per day.

**Table 1:** Baseline clinical characteristics of patients

	NG110 (*n* = 49)	NG120 (*n* = 50)	NG130 (*n* = 50)	WG (*n* = 38)	*p*
Age (years)	59 ± 10	63 ± 8	61 ± 7	63 ± 8	0.224
Male	27 (55.1%)	36 (72%)	32 (64%)	21 (55.3%)	0.269
Weight (kg)	76 ± 13	74 ± 9	73 ± 9	68 ± 11	0.016
Nonparoxysmal AF	21 (42.9%)	19 (38%)	14 (28%)	26 (68.4%)	0.002
Coronary artery disease	10 (20.4%)	7 (14%)	4 (8%)	8 (21.1%)	0.259
Hypertension	24 (49%)	23 (46%)	28 (56%)	18 (47.4%)	0.763
Diabetes mellitus	6 (12.2%)	8 (16%)	10 (20%)	7 (18.4)	0.754
Heart failure	5 (10.2%)	2 (4%)	2 (4%)	5 (13.2)	0.257
Stroke/TIAs	4 (8.2%)	3 (6%)	4 (8%)	4 (10.5%)	0.896
CHADS_2_ score	0.9 ± 0.8	0.8 ± 0.8	0.9 ± 0.8	0.9 ± 0.9	0.855
0	19 (38.8%)	21 (42%)	19 (38%)	15 (39.5%)	0.863
1	18 (36.7%)	20 (40%)	18 (36%)	11 (28.9%)	
≥ 2	12 (24.5%)	9 (18%)	13 (26%)	12 (31.6%)	
CHA_2_DS_2_-VASc score	1.8 ± 1.2	1.7 ± 1.3	1.7 ± 1.2	2.1 ± 1.6	0.429
HAS-BLED score	0.9 ± 0.9	0.9 ± 0.8	0.9 ± 0.7	1.0 ± 0.9	0.941
LAD (mm)	40 ± 6	41 ± 5	41 ± 6	42 ± 6	0.338
LVD (mm)	40 ± 6	47 ± 4	47 ± 4	46 ± 6	0.000
LVEF (%)	55 ± 9	57 ± 5	56 ± 5	55 ± 8	0.433
BNP (pg/mL)	176.6 ± 220.2	316.7 ± 396.8	179.9 ± 166.8	597.6 ± 753.2	0.261
Ccr (mL/min)	99 ± 19	93 ± 18	90 ± 23	90 ± 19	0.118
INR	1.1 ± 0.2	1.0 ± 0.1	1.0 ± 0.1	2.2 ± 0.9	0.000
TTR (%)	0	0	0	63.16	0.000
Thrombocyte (10^9^/L)	187 ± 47	203 ± 45	205 ± 49	206 ± 64	0.246
Hemoglobin (g/L)	145 ± 18	148 ± 18	145 ± 18	140 ± 17	0.218
Leukocyte (10^9^/L)	6.2 ± 1.6	6.4 ± 1.7	6.1 ± 1.5	5.9 ± 1.8	0.564
TG (mmol/L)	1.3 ± 0.6	1.5 ± 0.7	1.8 ± 1.8	1.7 ± 1.7	0.376
HDL (mmol/L)	1.2 ± 0.3	1.1 ± 0.2	1.2 ± 0.3	1.1 ± 0.2	0.084
LDL (mmol/L)	2.5 ± 1.5	2.3 ± 0.7	2.5 ± 0.6	2.3 ± 0.7	0.703
TC (mmol/L)	4.4 ± 1.0	4.3 ± 0.9	4.7 ± 1.0	4.3 ± 0.9	0.211
Anticoagulants (*n*)					0.619
Dabigatran	13 (26.5%)	18 (36%)	17 (34%)	0 (0)	
Rivaroxaban	36 (73.5%)	32 (64%)	33 (66%)	0 (0)	
Procedure time (min)	140 ± 42	131 ± 39	132 ± 38	138 ± 34	0.582
X-ray dose (msv)	9 ± 4	10 ± 3	11 ± 5	13 ± 11	0.186
Ablation time (min)	104 ± 37	99 ± 31	99 ± 34	104 ± 32	0.779

*TIAs* transient ischemic attacks, *LAD* left atrial diameter, *LVD* left ventricular diameter, *LVEF* left ventricular ejection fraction, *BNP* brain natriuretic peptide, *Ccr* creatinine clearance rate, *INR* international normalized ratio, *TTR* time in range, *TG* triglyceride, *HDL* high density lipoprotein, *LDL* low density lipoprotein, *TC* total cholesterol

### Intraprocedural ACTs

**Fig. 3:**
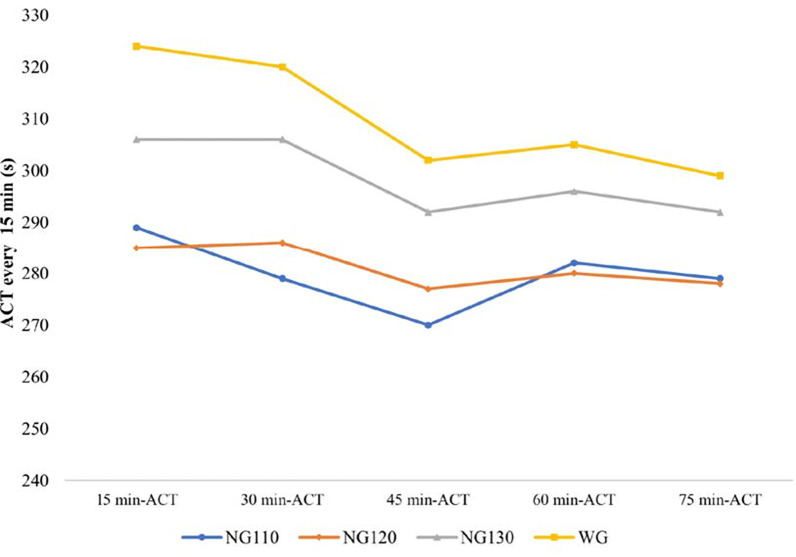
Intraprocedural ACTs of the four groups

There was no difference in baseline ACT between the WG and the NGs (NG110, NG120, NG130). The ACTs at each 15-min interval are shown in Fig. 3 and Table 2. The 15 min-ACT, 30 min-ACT, and 45 min-ACT were significantly longer in the WG than in NG110 and NG120. There was no significant difference in 60 min-ACT and 75 min-ACT between the WG and NG110, or between the WG and NG120. Moreover, there was no significant difference in all ACTs between the WG and NG130. There was 23.9% of ACTs in the WG were higher than 350 s, which was significantly higher than NGs (Additional file 1: Additional file 3, Table 2).

**Table 2:** Intraprocedural ACTs and the percentage of measurements at the target ACT

ACT (s)	WG	NG110	*p*	WG	NG120	*p*	WG	NG130	*p*
Baseline ACT	137 ± 44	128 ± 46	0.450	137 ± 44	127 ± 45	0.352	137 ± 44	129 ± 34	0.432
15 min-ACT	324 ± 65	289 ± 51	0.015	324 ± 65	285 ± 49	0.006	324 ± 65	306 ± 51	0.182
30 min-ACT	320 ± 61	279 ± 41	0.001	320 ± 61	286 ± 26	0.003	320 ± 61	306 ± 44	0.227
45 min-ACT	302 ± 58	270 ± 39	0.010	302 ± 58	277 ± 32	0.037	302 ± 58	292 ± 37	0.389
60 min-ACT	305 ± 72	282 ± 32	0.112	305 ± 72	280 ± 28	0.089	305 ± 72	296 ± 31	0.526
75 min-ACT	299 ± 55	279 ± 47	0.179	299 ± 55	278 ± 29	0.094	299 ± 55	292 ± 26	0.555
Percentage of measurements at the target ACT (%)	63.4 ± 36.2	73.4 ± 32.6	0.179	63.4 ± 36.2	82.2 ± 23.6	0.007	63.4 ± 36.2	84.8 ± 23.7	0.003

*ACT* activated clotting time

### The percentage of measurements at the target ACT

**Fig. 4:**
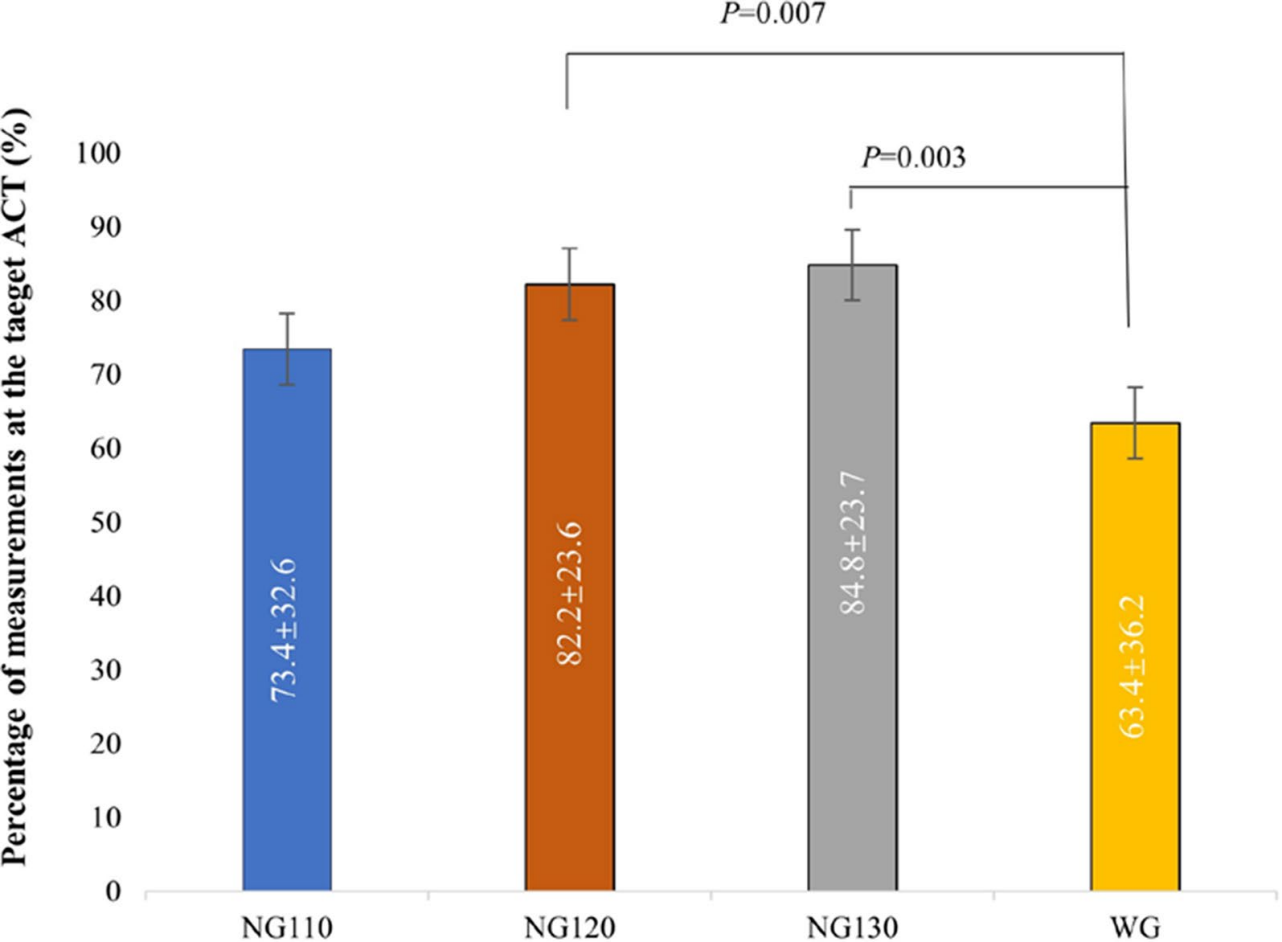
Comparison of the percentage of measurements at the target ACT between the WG and the NGs

**Fig. 5:**
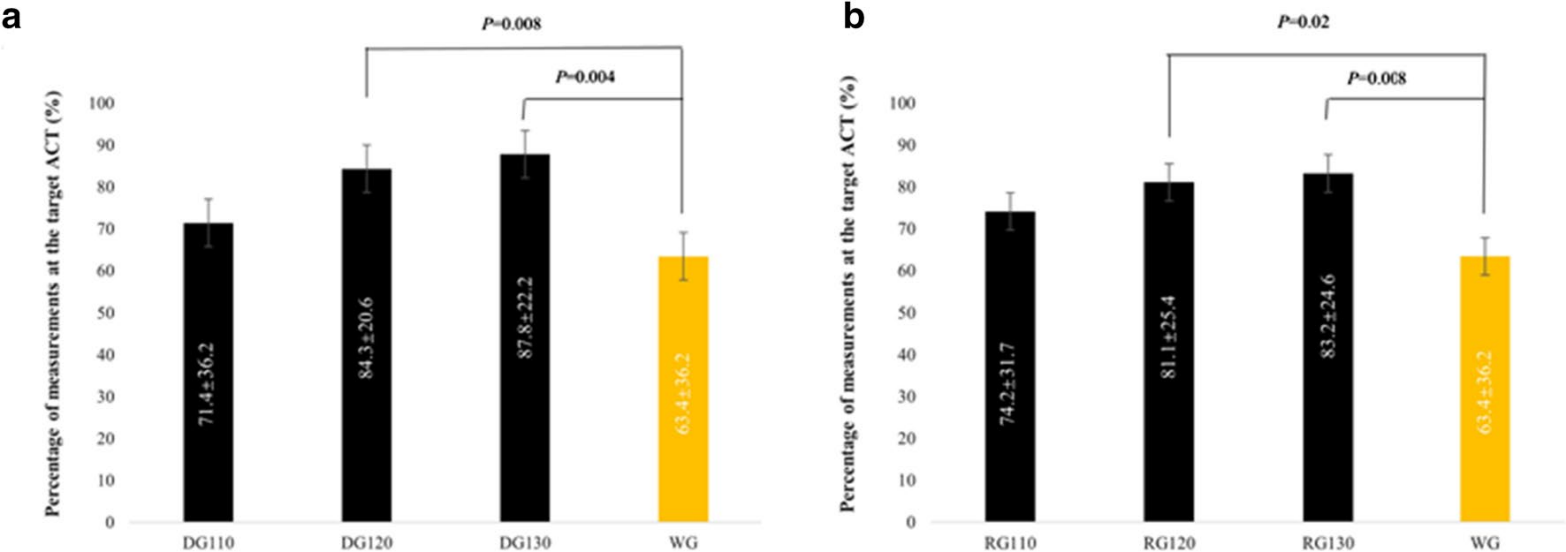
Comparison of the percentage of measurements at the target ACT between the DG and the WG, and between the RG and the WG

**Fig. 6:**
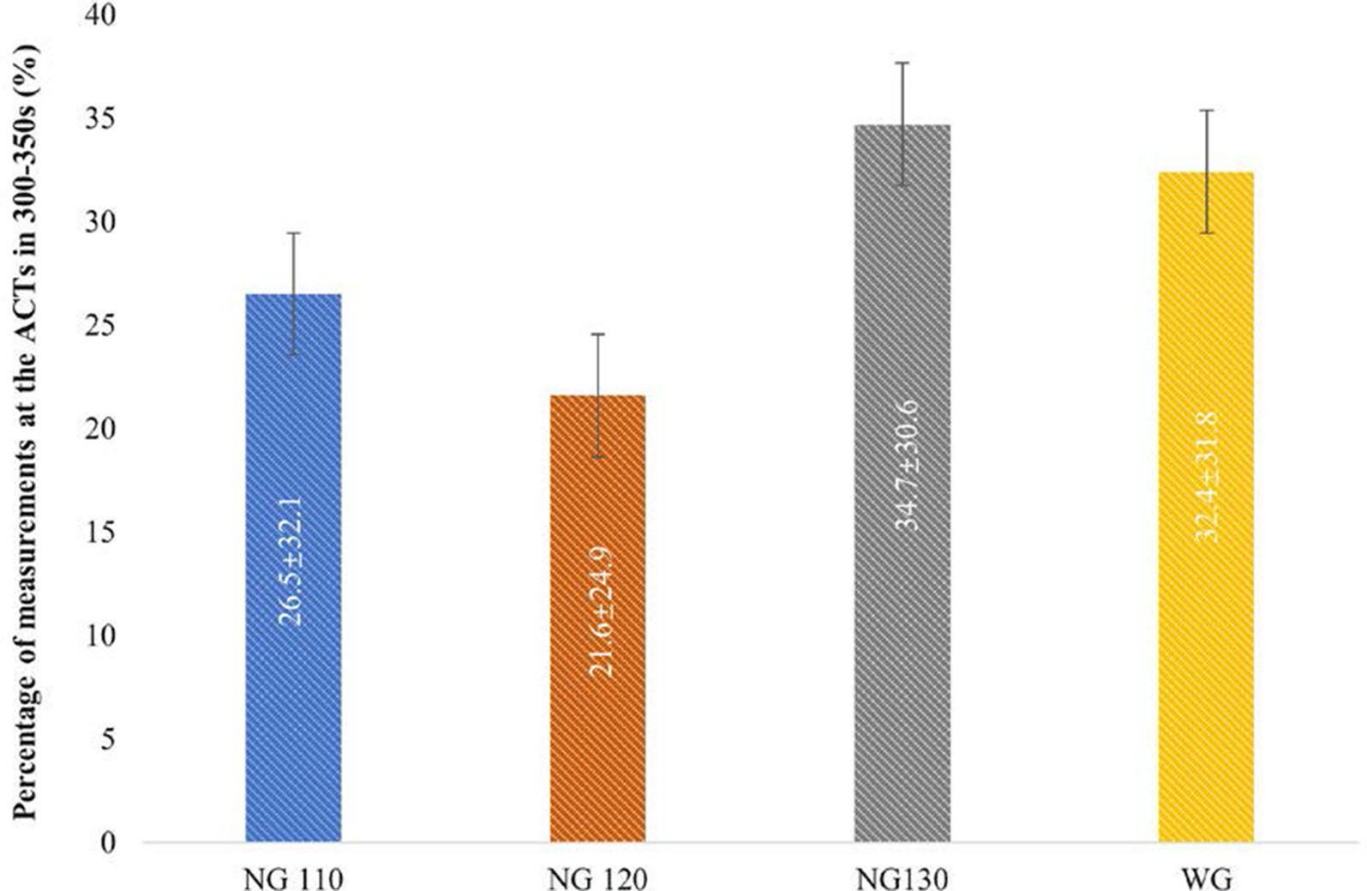
Comparison of the percentage of measurements at the ACTs in 300–350 s between the WG and the NGs

The average percentage of measurements at the target ACT in NG120 (82.2 ± 23.6%) and NG130 (84.8 ± 23.7%) were remarkably higher than that in WG (63.4 ± 36.2%, *p* = 0.007, 0.003, respectively). No significant difference was detected between NG110 and the WG (73.4 ± 32.6% vs. 63.4 ± 36.2%, *p* = 0.179), which is shown clearly in Fig. 4. These differences were irrelevant to the type of NOAC (dabigatran or rivaroxaban) in subgroup analysis, which compared the average percentage of measurements at the target ACT between the dabigatran groups (DG110, DG120, DG130) and the WG and between the rivaroxaban groups (RG110, RG120, RG130) and the WG (Fig. 5). The average percentage of measurements at the ACTs in 300–350 s between the WG and the three NGs were compared (Fig. 6 and Additional file 1: Additional file 4, Table 3). No significant difference was detected; however, the percentage of the ACTs in 300–350 s in the NG130 was higher than that in the WG (32.4 ± 31.8 vs. 34.7 ± 30.6, *p* = 0.735).

### Bleeding and thromboembolic complications

There was no significant difference in total bleeding or thromboembolic complications among the four groups (Table 3). In NG130, one major bleeding complication (blood transfusion required due to pericardial effusion) occurred in a paroxysmal AF patient who took dabigatran 110 mg twice a day. In addition to bilateral PVI, he also received a mitral annulus (5 cm from the coronary sinus ostium) ablation for the left accessory pathway, confirmed in the following electrophysiological study. The intraprocedural ACT values were between 304–334 s (mean ACT value was 318.8 s). The pericardiac effusion occurred at the end of the procedure, reduced after pericardial drainage and intraoperative blood transfusion, and then disappeared gradually within 48 h after the procedure. Two minor bleeding complications (one pericardial effusion resolved spontaneously, one inguinal hematoma with no further treatment) in the WG were observed.

**Table 3:** Bleeding and thromboembolic complications

Complications (*n*)	NG110	NG120	NG130	WG	*p*
Thromboembolic complications (*n*)	0 (0)	0 (0)	0 (0)	0 (0)	N/A
Stroke/TIAs	0 (0)	0 (0)	0 (0)	0 (0)	N/A
DVT	0 (0)	0 (0)	0 (0)	0 (0)	N/A
PE	0 (0)	0 (0)	0 (0)	0 (0)	N/A
Bleeding complications (*n*)	1 (2%)	1 (2%)	2 (4%)	2 (5%)	0.849
Major bleeding	0 (0)	0 (0)	1 (2%)	0 (0)	1.000
Cardiac tamponade	0 (0)	0 (0)	0 (0)	0 (0)	N/A
Delayed cardiac tamponade	0 (0)	0 (0)	0 (0)	0 (0)	N/A
Retroperitoneal hemorrhage	0 (0)	0 (0)	0 (0)	0 (0)	N/A
Hemoglobin drop ≥ 4 g/dL	0 (0)	0 (0)	0 (0)	0 (0)	N/A
Blood transfusion required	0 (0)	0 (0)	1 (2%)	0 (0)	1.000
Minor bleeding	1 (2%)	1 (2%)	1 (2%)	2 (5%)	0.812
Pericardial effusion	0 (0)	0 (0)	0 (0)	1 (2.5%)	0.203
Inguinal hematoma	1 (2%)	1 (2%)	1 (2%)	1 (2.5%)	1.000
Hematuria	0 (0)	0 (0)	0 (0)	0 (0)	N/A
Bleeding/thromboembolic complications	1 (2%)	1 (2%)	2 (4%)	2 (5%)	0.849

*TIAs* transient ischemic attacks, *DVT* deep venous thrombosis, *PE* pulmonary embolism

## Discussion

This study demonstrated that for AF patients receiving NOACs, an initial heparin dosage of 120 U/kg or 130 U/kg could provide adequate intraprocedural anticoagulation with a favorable safety profile, regardless of whether the NOAC was dabigatran or rivaroxaban.

Compared with warfarin, NOACs are noninferior in the prevention of strokes or systemic embolism and have a favorable safety profile; in addition, it is easier for patients adhere to NOAC treatment than to warfarin treatment [7]. This superiority was more evident in patients with poor INR control (TTR < 66%) or in Asians [8, 9]. In our study, there was an interesting phenomenon in which the WG's body weight was lower than that in the NG, indicating that patients with high weight were more inclined to choose NOACs for adequate adherence [10]. Indeed, none of the patients enrolled were underweight, and the intraoperative heparin dose was based on body weight.

For AF patients undergoing RFCA, the increased risk of periprocedural thromboembolic and bleeding complications is an intractable issue. The occurrence of thromboembolism during RFCA may be due to multiple factors, including catheter manipulation, especially in the left atrium; endothelial injury; the formation of necrotic debris; hypercoagulability; the activation of coagulation triggered by intrinsic or extrinsic pathways; and altered blood flow after AF conversion [11]. Subsequent atrial stunts and decreased contractility followed by ablation procedures may be attributed to postprocedural thromboembolism [12]. Asymptomatic cerebral infarction may be a common thromboembolic event and is associated with the number of intraprocedural electrical cardioversions [13]. According to present studies and recent guidelines, it is preferable to administer therapeutic oral anticoagulants (OAC) for at least three weeks before ablation and two months after ablation and continue long-term use for patents with stroke risk (CHA_2_DS_2_-VASc score ≥ 2 in men or ≥ 3 in women) [14, 15]. Uninterrupted periprocedural anticoagulant treatment is superior to the interrupted strategy with intravenous heparin or subcutaneous low-molecular-weight heparin (LMWH) [16, 17]. Furthermore, uninterrupted NOAC treatment surpass warfarin treatment [18, 19]. Whether it is necessary to omit one or two NOAC doses before ablation is indeterminate. The regimens of the omission of one or two NOAC doses before ablation was not recommended in the 2020 ESC guidelines for AF because OAC treatment was truly uninterrupted in the related RCTs [14]. Gorla et al. [20] compared three strategies for periprocedural OAC administration: uninterrupted, mildly interrupted (< 12 h), and interrupted (> 12 h) and found no difference in the number of major bleeding and thromboembolic complications. However, the weighted mean incidence of overall bleeding was higher in the uninterrupted and mildly interrupted strategies than in the interrupted strategy, indicating that an interrupted approach may also be safe and effective.

A safe and adequate intraprocedural anticoagulation is essential during the ablation procedure, and a dose of heparin should be administered before or immediately following transseptal puncture. Then, heparin will be infused intravenously to achieve and maintain the target ACT [21]. Heparin's standard initial dosage was 50–100 U/kg, and 100 U/kg was more common for patients receiving warfarin [22]. For patients who are on NOACs, heparin's initial dosage is higher and these patients need more time to achieve the target ACT and more heparin to maintain their target ACT compared with patients anticoagulated with warfarin [23]. Yamaji et al. [24] found that adequate initial heparin dosages for AF ablation in patients on NOACs are 10–20% higher than those in patients on warfarin, and they indicated that 120 or 130 U/kg dabigatran, and 130 U/kg rivaroxaban and apixaban are adequate initial heparin dosages. These findings are consistent with ours. The ACTs in WG were higher and achieved the target ACT earlier than NGs. Moreover, more ACTs in WG were higher than 350 s, which was over the target ACT, resulting in a lower percentage of measurements at the target ACT. Kishima et al. [5] developed a new initial heparin administration regimen with heparin added every 30 min based on the measured ACT to raise the target ACT (≥ 300 s) achievement rate in patients on NOACs. For patients with baseline ACT > 130 s, an initial dose of “100U/kg + 3000U” was administrated, followed by intermittent intravenous heparin. For patients with a baseline ACT < 130 s, when the initial heparin dose was increased to “100 U/kg + 5000 U” from “100 U/kg + 3000 U”, the rate of achieving the target ACT rose to 80.5% from 41.6% with no increase in the number of bleeding complications.

Currently, there is no consensus on the optimal intraoperative target ACT value. As mentioned above, AF-related guidelines and recommendations advocate maintaining an ACT of 300–350 s; however, a lower target ACT (< 300 s) was evaluated in some studies [22, 25]. Some authors have shown that RFCA of AF could be performed under lower intraoperative target ACT values with no increased risk of stroke or thromboembolic events and reduced bleeding complications in their studies [26, 27]. According to our experiences and previous Chinese guideline [4], the target ACT in this study was set as 250–350 s. There was no thromboembolic complication detected in this study; thus, we think a conservative ACT target is reasonable based on more frequent ACT monitoring and the wide use of the saline-irrigated catheter. According to the recently widely accepted guideline, we compared the average percentage of ACTs in 300–350 s [21, 28]. No significant difference was detected; however, the percentage in the NG130 was higher than that in the WG, indicating the dosage of 130 U/kg can provide a more adequate intraprocedural anticoagulant effect.

Some researchers suggested that a continuous heparin intravenous infusion is superior to an intermittent infusion for maintaining a target ACT during the AF ablation [29]. The percentage of ACT measurements at the optimal ACT in continuous heparin administration was significantly higher than that in the intermittent strategy, with lower total heparin levels and more stable ACT levels. This might be due to the dose-dependent pharmacokinetics and short half-life of heparin [30]. In our study, the administration of intravenous heparin after the initial administration was discontinuous, and the dosage of additional heparin was based on the measured ACT value, which was monitored every 15 min. There was no increase in the incidence of bleeding or thrombotic complications. Furthermore, CA of AF is a well-established treatment, and the CA procedure can be completed within 120 min, especially when performed by well-trained operators. Thus, we consider that intermittent intraprocedural heparin infusion guided by more frequent ACT monitoring is feasible and safe.

The average total heparin dose used to manage ACT was slightly higher, and the mean ACT level was marginally lower in rivaroxaban than in warfarin in the VENTURE-AF trial [17]. This subtle difference in ACTs may vary with the selected NOAC. Kottmaier et al. [31] found that the baseline ACT and mean ACT were higher in patients who received uninterrupted edoxaban than in those who received warfarin. This reason is unclear and may be related to the small sample number in the edoxaban group and the conservative target ACT (280–300 s). We also found that the intraprocedural ACT levels were higher in the WG than in the NGs. However, no significant difference was detected between the WG and NG130, which may be due to a higher initial dose of heparin in NG130. The baseline ACT in the WG was higher than that in the NGs, but there was no significant difference in our study.

Bleeding complications of RFCA are infrequent; access-site hematoma, followed by pericardial effusion with tamponade, are common among them [32]. The incidence of hemorrhage in our study was approximately 2–5%, which is similar to that reported in previous research. The incidence of bleeding was slightly higher in the WG than in the NGs, especially for those who received a lower initial dosage of heparin. Although one patient occurred pericardiac effusion in NG130, the incidence was still low and might be due to the additional ablation (alation at mitral annulus). Notably, the ACTs in NG130 achieved the target earlier than in NG110 and NG120. Compared with warfarin, there was a trend favoring NOACs in terms of major bleeding complications during the ablation of AF [33].

## Limitations

This study is a single-center study with a small sample. There was no significant difference in the incidence of perioperative bleeding or thromboembolic complications among the four groups; therefore, we could not fully assess the safety and risk. The target ACT in this study is slightly lower than the latest guideline. Moreover, we only compared the anticoagulation effect of dabigatran at 110 mg in this study, and this does not indicate the effect of the dose of 150 mg dabigatran. Multicenter trials with a large sample are needed to verify this hypothesis further.

## Conclusion

The initial heparin dosage and total heparin were higher for patients with AF on uninterrupted NOAC treatment who were undergoing RFCA than for patients taking warfarin. The appropriate initial heparin dosage was 120 U/kg or 130 U/kg for patients anticoagulated with NOACs. The dosage of 130 U/kg allowed ACT to reach the target earlier than the dosage of 120 U/kg.

## Supplementary Information

**Additional file 1.** Appropriate intraprocedural initial heparin dosing in patients undergoing catheter ablation for atrial fibrillation receiving uninterrupted non-vitamin K antagonist oral anticoagulant treatment.

## Data Availability

The datasets generated and/or analysed during the current study are not publicly available due to data protection but are available from the corresponding author on reasonable request.

## Abbreviations

ACT: Activated clotting time
AF: Atrial fibrillation
AFL: Atrial flutter
CA: Catheter ablation
DG: Dabigatran group
LAD: Left atrial diameter
LVEF: Left ventricular ejection fraction
NG: NOAC group
NOAC: Non-vitamin-K antagonist oral anticoagulant
INR: International normalized ratio
OAC: Oral anticoagulants
PV: Pulmonary vein
TTR: Time in range
RCT: Randomized controlled trials
RFCA: Radiofrequency catheter ablation
RG: Rivaroxaban group
SD: Standard deviation
TIAs: Transient ischemic attacks
WG: Warfarin group
