# Supplementary material for: Appropriate intraprocedural initial heparin dosing in patients undergoing catheter ablation for atrial fibrillation receiving uninterrupted non-vitamin-K antagonist oral anticoagulant treatment
Source: BMC Cardiovasc Disord. 2021 Apr 27;21:214. doi: 10.1186/s12872-021-02032-3 (PMC8077881; doi:10.1186/s12872-021-02032-3)
Supplement: Supplementary file 1 — Additional file 1. Appropriate intraprocedural initial heparin dosing in patients undergoing catheter ablation for atrial fibrillation receiving uninterrupted non-vitamin K antagonist oral anticoagulant treatment. [file 12872_2021_2032_MOESM1_ESM.docx]

**Title page**

**(Additional file)**

**Type of manuscript:** Research article.

**Article title:** Appropriate Intraprocedural Initial Heparin Dosing in Patients Undergoing Catheter Ablation for Atrial Fibrillation Receiving Uninterrupted Non-Vitamin-K Antagonist Oral Anticoagulant Treatment.

**List of Authors**: Rong-feng Zhang^1#^, Cheng-ming Ma^1#^, Na Wang^2#^, Ming-hui Yang^1#^, Wen-wen Li^1^, Xiao-meng Yin^1^, Ying-xue Dong^1^, Xiao-hong Yu^1^, Xian-jie Xiao^1^, Yun-long Xia^1*^, Lian-jun Gao^1*^

**The affiliation of the authors:**

1. Department of Cardiology, Institute of Cardiovascular Diseases, First Affiliated Hospital of Dalian Medical University, Dalian, China
2. Department of Ultrasonics, Second Affiliated Hospital of Xi’an Jiaotong University, Xi’an, China.

This manuscript has been read and approved by all the authors, and each author believes that the manuscript represents honest work.

**Corresponding author:** Yun-long Xia and Lian-jun Gao.

Address: Department of Cardiology, Institute of Cardiovascular Diseases, First Affiliated Hospital of Dalian Medical University, 193# Lianhe Road, Shahekou District, Dalian, China.

Phone: +86 18098875777.

Email: lianjun_gaophd@163.com

**Grant support:** This work was supported by the National Natural Science Foundation of China (No. 81800294).

**Acknowledgment:** We thank AJE (https://www.aje.cn) for its linguistic assistance during the preparation of this manuscript.

**Conflict of interest:** None declared.

1. **The inclusion/ exclusion criteria**

The inclusion criteria were as follows: (1) patients aged 18-75 years with symptomatic or asymptomatic AF that was documented by surface electrocardiograph (ECG); (2) international standardized ratio (INR) of patients receiving warfarin was between 2.0-3.0 and the Time in Therapeutic Range (TTR) >60%; patients with NOACs were taken the routine dose (dabigatran for 110mg twice a day, rivaroxaban 15mg or 20mg for once a day). (3) accorded with the indication of catheter ablation without catheter ablation related contraindication and signed the informed consent.

The exclusion criteria were as follows: (1) patients with a past medical history of cardiac surgery or cardiac device implantation; (2) left ventricular ejection fraction (LVEF) ≤40% or left atrial diameter (LAD) ≥55mm; (3) severe hepatic or renal insufficiency (creatinine clearance rate ≤30 ml/min or glutamate transaminase/glutamate transaminase ≥3); (4) severe coagulation disorders; (5) life expectancy ≤ 1 year.

1. **Supplementary Table 1. Baseline clinical characteristics of dabigatran and rivaroxaban group.**

|  | DG | | | | | RG | | | |  |
| --- | --- | --- | --- | --- | --- | --- | --- | --- | --- | --- |
|  | 110U/Kg | 120U/Kg | 130U/Kg | *P* | 110U/Kg | | 120U/Kg | 130U/Kg | *P* |  |
| Age (years) | 57±11 | 61±8 | 60±8 | 0.415 | 60±9 | | 63±8 | 62±7 | 0.306 |  |
| Male | 9(69.2%) | 17(94.4%) | 13(76.6%) | 0.169 | 18(50%) | | 19(59.4%) | 19(57.6%) | 0.746 |  |
| Weight (kg) | 77±12 | 78±9 | 74±10 | 0.565 | 76±14 | | 73±9 | 73±8 | 0.330 |  |
| Non-paroxysmal AF | 7(53.8%) | 12(66.7%) | 14(82.4%) | 0.285 | 22(61.1%) | | 19(59.4%) | 22(66.7%) | 0.871 |  |
| CAD | 0(0%) | 1(5.6%) | 13(76.5%) | 1.000 | 10(27.8%) | | 4(12.5%) | 3(9.1%) | 0.08 |  |
| Hypertension | 8(61.5%) | 5(27.8%) | 8(47.1%) | 0.165 | 16(44.4%) | | 18(56.3%) | 20(60.6%) | 0.391 |  |
| Diabetes mellitus | 2(15.4%) | 2(11.1%) | 3(17.6%) | 0.882 | 4(11.1%) | | 6(18.8%) | 7(21.2%) | 0.503 |  |
| Heart failure | 2(15.4%) | 1(5.6%) | 1(5.9%) | 0.663 | 3(8.3%) | | 1(3.1%) | 1(3.0%) | 0.616 |  |
| Stroke/TIA | 2(15.4%) | 2(11.1%) | 2(11.8%) | 1.000 | 2(5.6%) | | 1(3.1%) | 2(6.1%) | 1.000 |  |
| CHAD_2_ score | 1.7±1.2 | 1.3±0.9 | 1.3±1.2 | 0.536 | 1.9±1.2 | | 2.0±1.5 | 1.9±1.2 | 0.909 |  |
| 0 | 4(30.8%) | 8(44.4%) | 7(41.2%) | 0.376 | 15(41.7%) | | 13(40.6%) | 12(36.4%) | 0.992 |  |
| 1 | 6(46.2%) | 9(50%) | 5(29.4%) |  | 13(36.1%) | | 11(34.4%) | 13(39.4%) |  |  |
| >2 | 3(23.1%) | 1(5.6%) | 5(29.4%) |  | 8(22.2%) | | 8(25%) | 8(24.2%) |  |  |
| CHA_2_DS_2_-VASc score | 1.1±0.9 | 0.6±0.6 | 0.8±0.2 | 0.277 | 0.8±0.8 | | 0.9±0.9 | 0.9±0.9 | 0.801 |  |
| HAS-BLED score | 0.9±1.0 | 0.6±0.8 | 0.6±0.8 | 0.574 | 0.0±0.8 | | 1.1±0.8 | 1.0±0.7 | 0.358 |  |
| LAD (mm) | 41±6 | 43±6 | 42±7 | 0.808 | 40±6 | | 40±5 | 40±5 | 0.927 |  |
| LVD (mm) | 48±6 | 47±5 | 48±4 | 0.754 | 49±5 | | 47±3 | 47±4 | 0.174 |  |
| LVEF (%) | 54±10 | 55±7 | 56±5 | 0.720 | 55±8 | | 57±2 | 56±5 | 0.194 |  |
| BNP (pg/mL) | 50.8±31.7 | 143.3±97.2 | 110.1±56.7 | 0.283 | 223.7±244.3 | | 420.8±484.5 | 207.9±190.0 | 0.388 |  |
| Ccr(mL/min) | 94±17 | 91±15 | 88±25 | 0.728 | 100±20 | | 94±19 | 91±22 | 0.217 |  |
| INR | 1.1±0.1 | 1.1±0.1 | 1.0±0.1 | 0.121 | 1.1±0.2 | | 1.0±0.1 | 1.0±0.1 | 0.180 |  |
| Thrombocyte(10^9^/L) | 191±41 | 201±49 | 204±58 | 0.770 | 186±50 | | 204±44 | 206±44 | 0.154 |  |
| Hemoglobin (g/L) | 155±23 | 153±18 | 146±20 | 0.467 | 141±14 | | 145±18 | 143±17 | 0.584 |  |
| Leukocyte (10^9^/L) | 6.3±1.4 | 7.1±1.6 | 6.3±1.6 | 0.254 | 6.2±1.7 | | 6.0±1.6 | 6.0±1.4 | 0.827 |  |
| TG (mmol/L) | 1.3±0.6 | 1.4±0.7 | 1.8±1.3 | 0.342 | 1.4±0.7 | | 1.5±0.7 | 1.8±2.1 | 0.366 |  |
| HDL (mmol/L) | 1.2±0.3 | 1.2±0.2 | 1.2±0.4 | 0.999 | 1.2±0.3 | | 1.0±0.2 | 1.2±0.3 | 0.015 |  |
| LDL (mmol/L) | 2.6±0.9 | 2.4±0.6 | 2.5±0.7 | 0.895 | 2.5±1.7 | | 2.3±0.7 | 2.5±0.6 | 0.684 |  |
| TC (mmol/L) | 4.8±1.4 | 4.5±1.0 | 4.7±1.0 | 0.682 | 4.3±0.8 | | 4.2±1.0 | 4.7±1.0 | 0.132 |  |

1. **Supplementary Table 2. Percentage of Intraprocedural ACTs > 350s**

|  | WG | NG110 | *P* | WG | NG120 | *P* | WG | NG130 | *P* |
| --- | --- | --- | --- | --- | --- | --- | --- | --- | --- |
| Percentage of ACTs > 350s (%) | 23.9±13.7 | 3.9±15.4 | 0.001 | 23.9±13.7 | 3.6±12.2 | 0.001 | 23.9±13.7 | 5.3±16.4 | 0.003 |

1. **Supplementary Table 3. Percentage of measurements at the ACTs in 300-350s**

|  | WG | NG110 | *P* | WG | NG120 | *P* | WG | NG130 | *P* |
| --- | --- | --- | --- | --- | --- | --- | --- | --- | --- |
| Percentage of measurements at the ACTs in 300-350s (%) | 32.4±31.8 | 26.5±32.1 | 0.395 | 32.4±31.8 | 21.6±24.9 | 0.077 | 32.4±31.8 | 34.7±30.6 | 0.735 |
